# Robust Antigen Specific Th17 T Cell Response to Group A Streptococcus Is Dependent on IL-6 and Intranasal Route of Infection

**DOI:** 10.1371/journal.ppat.1002252

**Published:** 2011-09-22

**Authors:** Thamotharampillai Dileepan, Jonathan L. Linehan, James J. Moon, Marion Pepper, Marc K. Jenkins, Patrick P. Cleary

**Affiliations:** 1 Department of Microbiology, University of Minnesota, Minneapolis, Minnesota, United States of America; 2 Department of Microbiology-Center for Immunology, Medical School, University of Minnesota, Minneapolis, Minnesota, United States of America; 3 The Center for Immunology & Inflammatory Diseases, Division of Rheumatology, Allergy and Immunology, Massachusetts General Hospital, Cambridge, Massachusetts, United States of America; Harvard Medical School, United States of America

## Abstract

Group A streptococcus (GAS, *Streptococcus pyogenes*) is the cause of a variety of clinical conditions, ranging from pharyngitis to autoimmune disease. Peptide-major histocompatibility complex class II (pMHCII) tetramers have recently emerged as a highly sensitive means to quantify pMHCII-specific CD4^+^ helper T cells and evaluate their contribution to both protective immunity and autoimmune complications induced by specific bacterial pathogens. In lieu of identifying an immunodominant peptide expressed by GAS, a surrogate peptide (2W) was fused to the highly expressed M1 protein on the surface of GAS to allow in-depth analysis of the CD4^+^ helper T cell response in C57BL/6 mice that express the I-A^b^ MHCII molecule. Following intranasal inoculation with GAS-2W, antigen-experienced 2W:I-A^b^-specific CD4^+^ T cells were identified in the nasal-associated lymphoid tissue (NALT) that produced IL-17A or IL-17A and IFN-γ if infection was recurrent. The dominant Th17 response was also dependent on the intranasal route of inoculation; intravenous or subcutaneous inoculations produced primarily IFN-γ^+^ 2W:I-A^b+^ CD4^+^ T cells. The acquisition of IL-17A production by 2W:I-A^b^-specific T cells and the capacity of mice to survive infection depended on the innate cytokine IL-6. IL-6-deficient mice that survived infection became long-term carriers despite the presence of abundant IFN-γ-producing 2W:I-A^b^-specific CD4^+^ T cells. Our results suggest that an imbalance between IL-17- and IFN-γ-producing CD4^+^ T cells could contribute to GAS carriage in humans.

## Author Summary

Group A streptococcus (GAS) causes many different conditions, ranging from strep throat, flesh eating disease to post infectious complications involving the heart. Here, we used a novel technique to study the CD4^+^ T cell immune response against GAS infection in a mouse model. We first generated a recombinant GAS strain that expresses a specific epitope (2W) - M protein fusion and used this to intranasally inoculate mice. Peptide specific CD4^+^ T cells were concentrated and analyzed using 2W-MHC-II tetramers. This technology allowed us to probe the antigen specific CD4^+^ T cell response to new depths and certainty. Infection induced a robust 2W-specific Th17 cell response, which was dependent on the route of infection, IL-6, and was independent of superantigens. *IL-6^-/-^* mice were exquisitely susceptible to intranasal infection. However, those that survived became immune carriers, unable to clear streptococci from NALT. Further, multiple infections generated an IL-17^+^ IFN-γ^+^ double positive population of CD4^+^ T cells that are known to be associated with autoimmune disease in humans and directly responsible for autoimmune pathology in rodent models. Our results provide a new direction for understanding two important consequences of streptococcal pharyngitis, the very common immune carrier state, and the rarer state involving autoimmune complications.

## Introduction

Group A streptococcus (GAS, *Streptococcus pyogenes*) is an important bacterial pathogen that causes many different clinical conditions ranging from pharyngitis, impetigo, toxic shock syndrome, necrotizing fasciitis to post infectious autoimmune sequelae like acute rheumatic fever and glomerulonephritis [1], [2]. GAS is an important cause of morbidity and mortality worldwide. Estimates are that 500,000 deaths occur each year due to severe GAS infections [3]. GAS produces a wide variety of virulence factors that play important roles in adhesion, immune evasion, dissemination, tissue destruction and toxicity [1], [4]. Remarkably, one third of those treated with antibiotics continue to shed streptococci and a significant number of these carriers have recurrent disease by the same strain [5], [6]. Carrier state is an important public health problem because it maintains the cycle of disease in a community. Tonsils are known to harbor and shed streptococci, even after intense antibiotic therapy. Osterlund *et al* found that 93% of tonsils excised from children retained intracellular GAS, and others reported isolation of GAS from excised tonsils, confirming that this secondary lymphoid tissue is an important reservoir [7].

The capacity of GAS to survive in lymphoid tissue is not understood. Although GAS induces a humoral immune response in most individuals, streptococci can still persist. GAS also induces a CD4^+^ T cell response [8]. CD4^+^ T cells express T cell antigen receptors (TCR) that recognize short peptides bound to MHCII molecules (pMHCII) expressed by host antigen-presenting cells. During primary infection, the rare naïve CD4^+^ T cells, which by chance express TCRs complementary to bacterial pMHCII complexes, proliferate and differentiate into Th1, Th2, or Th17 effector cells that produce cytokines such as IFN-γ, IL-4 or IL-17, respectively, which help to eliminate the pathogen. Which of these effector cell types will develop during infection is determined by cytokines produced by cells of the innate immune system. For example in mice, the differentiation of Th17 cells *in vitro* depends on IL-6 and Transforming Growth Factor-beta (TGF-β) [9]. We recently demonstrated that GAS infection of the NALT, the murine equivalent of the tonsils, generates Th17 cells, which contribute to immune protection [10]. Lu *et al* have shown that Th17 cells protect against other streptococcal infections as well [11].

Previous studies used adoptive transfer of monoclonal TCR transgenic T cells to study T cell responses to infection with antigen tagged GAS [8], [12]. A drawback of this approach is that it results in an abnormally large number of naïve precursors, which experience inefficient activation due to competition for limiting pMHCII [13]-[15]. Therefore, the nature of the CD4^+^ T cell response to GAS infection under physiological conditions is still unknown.

To avoid these limitations, we used a new cell enrichment method based on fluorochrome-labeled pMHCII tetramers and magnetic beads [16] to characterize the endogenous polyclonal CD4^+^ T cell response to GAS. This approach depended on the availability of a tetramer of the I-A^b^ MHCII molecule of the preferred C57BL/6 mouse strain bound to a peptide from the GAS proteome. Unfortunately, no such peptide has been identified to date. We therefore produced a recombinant GAS strain that expresses an immunogenic peptide (EAWGALANWAVDSA) called 2W [17] fused to the M1 protein on its surface. 2W:I-A^b^ tetramer staining and magnetic bead enrichment was used to characterize 2W:I-A^b+^ CD4^+^ T cells from NALT and other lymphoid tissues after intranasal GAS-2W infection. Our results demonstrate that an intranasal infection is critical for mounting an effective IL-6-dependent pMHCII-specific Th17 response. A lack of this response led to a preponderance of Th1 cells and failure to control GAS infection. This work defines the Th17 response to GAS infection, and may shed light on the basis for the carrier state and autoimmune complications in humans.

## Results

### Generation of recombinant GAS strain that expresses the M1-2W fusion protein

**Figure 1:**
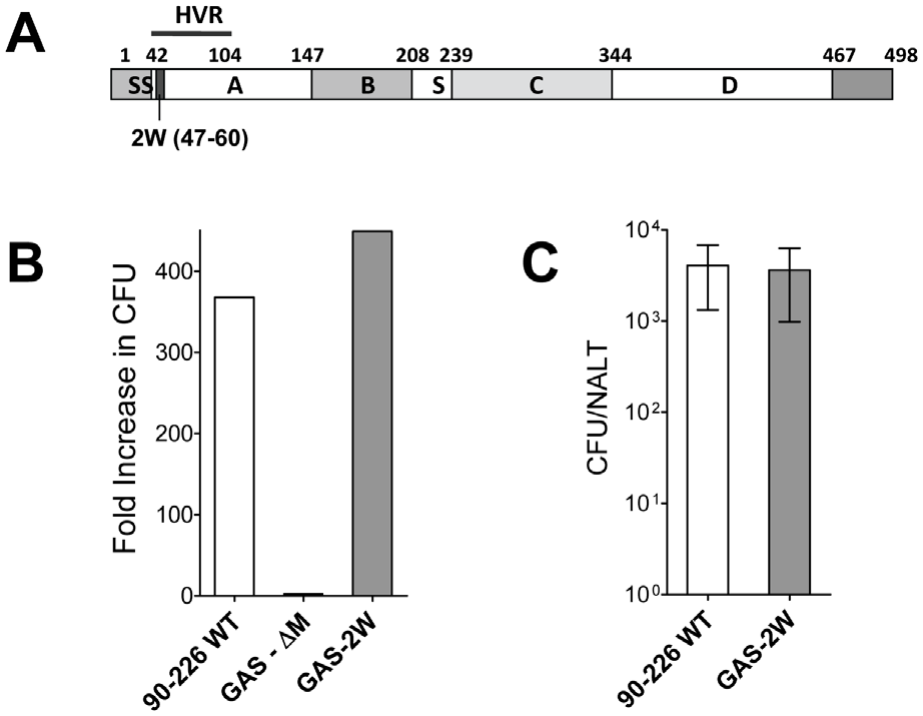
Construction of a Group A Streptococcus 90-226 strain that expresses the 2W (GAS-2W). (A) Schematic diagram of emm1.0::2W fusion protein that is expressed on the surface of GAS-2W. SS-Signal sequence; A - A region; B - B repeats; S - spacer region; C – C repeats; D – D region; HVR – Hyper-variable region; Cell wall anchor (466-496) (B) Survival and growth of strain GAS-2W in phagocytic human blood. 90-226 WT is the parent of GAS-2W and the M protein negative mutant GAS-ΔM. Fold increase was calculated as the number of CFU after 3 h in rotated blood divided by the initial number of CFU. Representative result from one of two independent experiments is shown. (C) Colonization of mouse NALT by intranasally administered Streptococci. Viable counts of 90-226-WT or GAS-2W streptococci recovered from homogenized NALT 24h following inoculation with 2×10^8^ CFU. The bars represent the mean ± SEM of four mice per group.

An M1 GAS strain 90–226 was genetically engineered to express the 14 amino acid 2W peptide (EAWGALANWAVDSA) as a cell wall surface hybrid M1 fusion protein. The hybrid emm1.0::2W gene was constructed in plasmid pFW5 in *E. coli* and then introduced into the chromosomal emm1.0 gene by allelic replacement [18]. The corresponding chimeric protein is composed of the 14 amino acid 2W peptide inserted in frame after the first five amino acids of the mature M1 surface protein (Fig. 1A). The strain, designated 90–226 emm1.0::2W (GAS-2W), is genetically stable without spectinomycin selection due to replacement of the wild-type gene in the chromosome. The anti-phagocytic property of the M1-2W fusion protein was assessed to test whether insertion of the 2W peptide altered the function of the M protein. GAS-2W was as resistant to phagocytosis as the wild-type 90-226 in whole blood bactericidal assays (Fig. 1B). An M^-^ variant 90-226 emm1.0::km (GAS-ΔM) [18] was included as a negative control and was susceptible to phagocytic killing as expected. Furthermore, the GAS-2W strain colonized mouse NALT as efficiently as the wild type (Fig. 1C).

### Intranasal infection with GAS-2W induced a strong antigen-specific Th17 response in C57BL/6 mice

**Figure 2:**
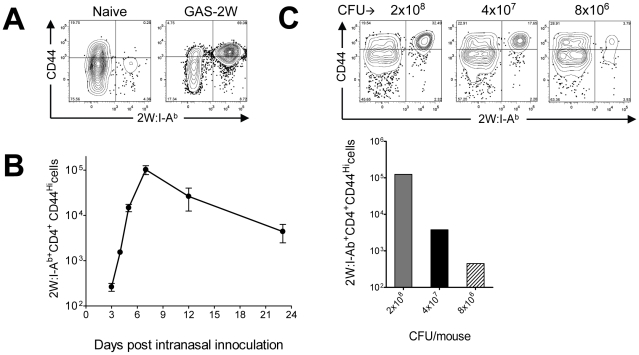
2W:I-A^b^-specific naïve CD4^+^ T cells expanded in response to intranasal infection with GAS-2W. (A) B6 mice were infected with GAS-2W (intranasal, 2×10^8^ CFU). Seven days later CD4^+^2W:I-A^b+^ cells were enriched from cervical lymph nodes and spleens and analyzed by flow cytometry. One representative of at least ten independent experiments is shown. CD44 indicates the number of antigen experienced CD4^+^ T cells (B) Change in the total number of 2W:I-A^b^-specific CD4^+^ T cells after a primary infection over time (n = 28). (C) Mean number of antigen specific CD4^+^2W:I-A^b+^ T cells is dose dependent. B6 mice were inoculated intranasally with 2×10^8^, 4×10^7^ and 8×10^6^ CFU/mouse, respectively. Seven days after infection CD4^+^2W:I-A^b+^ cells were isolated from cervical lymph nodes and spleen, and analyzed.

Spleen and lymph node cells from mice were pooled for quantification of 2W:I-A^b^-specific CD4^+^ T cells by pMHCII-based cell enrichment at various times after infection with GAS-2W. Each C57BL/6 mouse had between 200 and 250 2W:I-A^b^-specific CD4^+^ T cells before infection, and these cells expressed small amounts of CD44 (CD44^Lo^) on the surface as expected for naïve cells (Fig. 2A) [16], [19]. In mice that were inoculated intranasally with GAS-2W, this naïve 2W:I-A^b^-specific CD4^+^ T cell population expanded to ∼10^5^ cells in seven days (Fig. 2A). Most of these cells expressed large amounts of CD44 (CD44^Hi^), indicative of pMHCII-dependent activation. Expansion of 2W:I-A^b^-specific CD4^+^ T cells was first detected three days after primary infection with GAS-2W and increased rapidly to a peak seven days after inoculation (Fig. 2B). 2W:I-A^b^-specific CD4^+^ T cells then began to decline and reached 10% of the maximum level by day 20-post infection. Expansion of these CD4^+^ T cells was proportional to the dose of GAS-2W used for infection (Fig. 2C).

**Figure 3:**
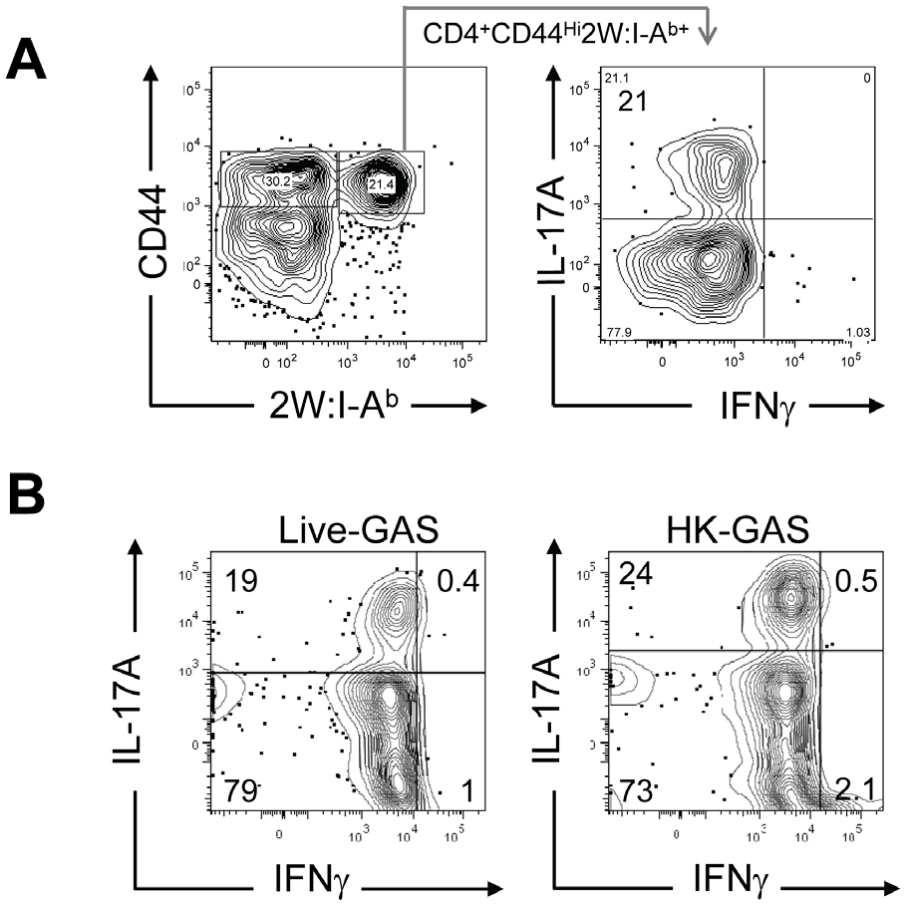
Primary intranasal GAS infection induces a Th17 response. Mice were inoculated once intranasally with 2×10^8^ CFU or equivalent numbers of heat-killed streptococci HK-GAS. (A) CD4^+^CD44^Hi^2W:I-A^b+^ cells enriched from spleens of mice that were inoculated with live GAS-2W and restimulated after a week in vivo by IV injection of heat killed GAS-2W (HK-GAS-2W). (B) CD4^+^CD44^Hi^2W:I-A^b+^ cells from mice that were inoculated intranasally with heat-killed GAS-2W and restimulated in vivo with HK-GAS-2W. One representative of three independent experiments is shown.

*In vivo* lymphokine production was measured by rechallenging mice with heat-killed GAS-2W (HK-GAS-2W) and then measuring intracellular lymphokines produced by 2W:I-A^b^-specific T cells 3 hours later [20]. 2W:I-A^b+^ cells in mice infected intranasally with live GAS-2W one week earlier produced IL-17A, but not IFN-γ after challenge with HK-GAS-2W (Fig. 3A). 2W:I-A^b+^ cells in mice inoculated intranasally with HK-GAS-2W one week earlier also produced IL-17A after challenge with HK-GAS-2W (Fig. 3B). Therefore, exposure of NALT to either live or dead GAS induced bacterial pMHCII-specific Th17 cells. In order to compare the approximate percentage of 2W:I-A^b^-specific Th17 cells to total GAS induced Th17 cells, B6 Mice were inoculated once intranasally with 2x10^8^ CFU of GAS-2W. Ten days after the infection mice were restimulated in vivo by IV injection of heat killed GAS-2W. 2W:I-A^b+^ CD4^+^ T cells from spleen were stained and enriched as described in the methods. Both bound (2W:I-A^b+^ CD4^+^ T cells) and unbound (2W:I-A^b-^ CD4^+^ T cells or flow through) fractions were collected and analyzed for intracellular cytokines IL-17A and IFN-γ. Total numbers of CD4^+^2W:I-A^b+^IL-17A^+^ and CD4^+^2W:I-A^b-^IL-17A^+^ cells were calculated for the entire spleen. The approximate ratio of 2W:I-A^b+^IL-17A^+^ cells to total 2W:I-A^b-^ IL-17A^+^ cells ranged from 1:7 to 1:12 (Fig S1).

**Figure 4:**
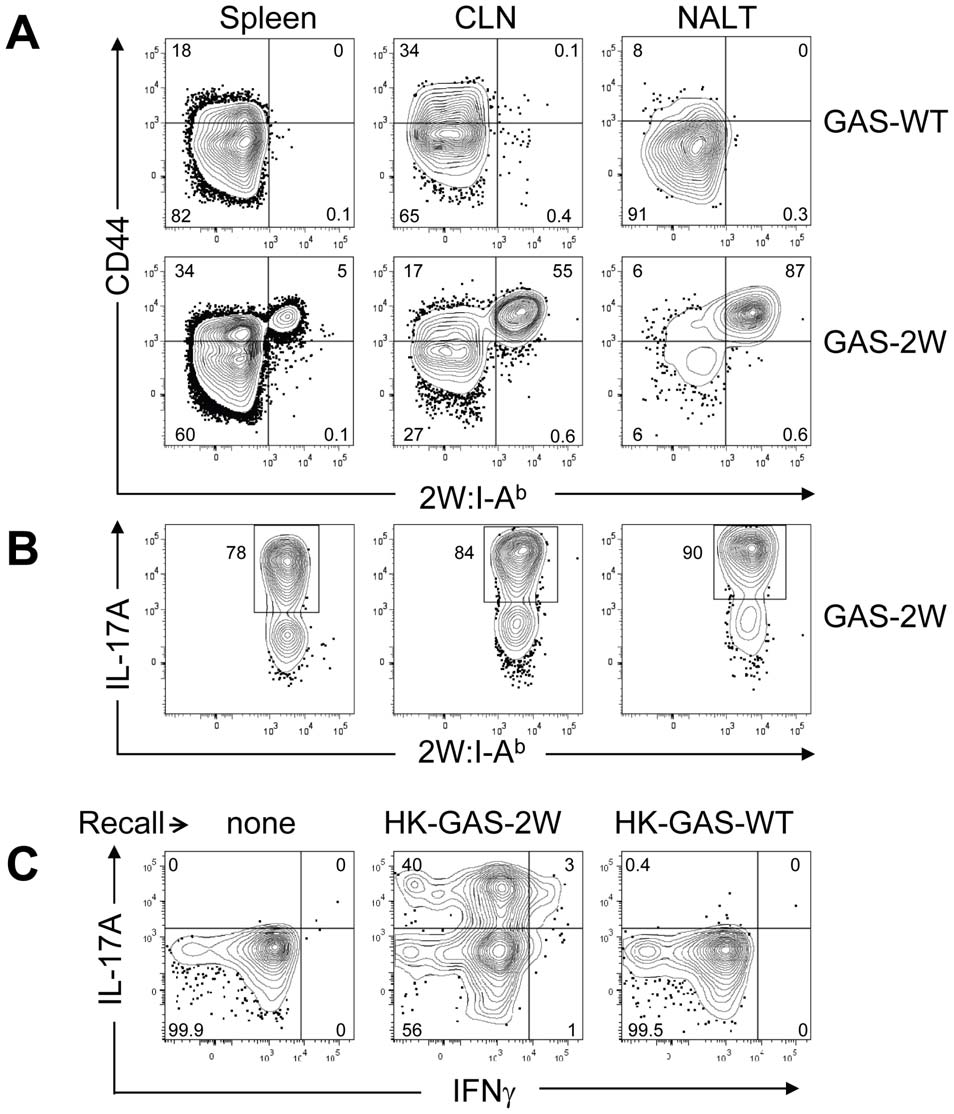
Clonal expansion of 2W:I-A^b^-specific CD4^+^ T cells and recall of IL-17A is antigen-specific and not due to Superantigens produced by GAS. (A) Tetramer enriched CD4^+^ T cells from Spleen, CLN and NALT of mice after three inoculations at weekly intervals. Top panel is from wild type GAS (GAS-WT) infected B6 mice and the lower panel is from B6 mice inoculated with (GAS-2W). (B) Analysis of IL-17A expression by CD4^+^ CD44^Hi^ 2W:I-A^b^-specific T cells in NALT, CLN and Spleen from the lower panel of part A. (C) Analysis of the specificity of IL-17A activation in CD4^+^ 2W:I-A^b+^ T cells originally primed by GAS-2W infection (twice). Primed mice were restimulated in vivo by IV injection of heat killed GAS-2W (HK-GAS-2W) or heat killed 2W negative GAS (HK-GAS-WT) 21 days after the last infection. CD4^+^CD44^Hi^2W:I-A^b+^ cells were isolated as described in methods and analyzed for IL-17A and IFN-γ expression.

Li *et al* reported that the Streptococcal superantigen, which contaminates commercial peptidoglycan preparations induced human lymphocytes to produce IL-17A [21]; therefore, experiments were performed to determine whether 2W:I-A^b^-specific CD4^+^ T cell clonal expansion was pMHCII-specific. B6 mice were inoculated three times at weekly intervals with either 2×10^8^ CFU of GAS-2W or wild type 2W^-^ GAS (GAS-WT). Three days after the last infection, mice were euthanized and enriched CD4^+^2W:I-A^b+^ T cells from spleen, cervical lymph nodes (CLN), and NALT were analyzed (Fig. 4.). Expansion of 2W:I-A^b^-specific CD4^+^ T cells only occurred in mice inoculated with GAS-2W (Fig. 4A), and not in mice inoculated with GAS-WT that lacked the 2W epitope. The number of CD4^+^2W:I-A^b+^ T cells in tissue from mice infected with GAS-WT was less than 200, similar to that of naïve mice. This indicates that the clonal expansion of 2W:I-A^b^-specific CD4^+^ T cells is a pMHCII-specific response and not due to superantigens produced by this strain of GAS (Fig. 4A). The same samples were restimulated *in vitro* with pharmacologic TCR mimics, PMA and ionomycin, and stained for intracellular cytokines in order to evaluate their cytokine phenotype. Most of the 2W:I-A^b^-specific CD4^+^ T cells from NALT, CLN and spleen produced IL-17A (Fig. 4B); greater than 75% of the 2W:I-A^b^-specific CD4^+^ T cells from all three tissues had a Th17 phenotype (Fig. 4B). The response was more robust in NALT, which is a preferred target of intranasal GAS infection. In NALT pMHCII-specific IL-17A producers reached more than 90% of the total 2W:I-A^b^-specific CD4^+^ T cells.

Even though initial priming and expansion of CD4^+^2W:I-A^b+^ T cells is independent of superantigen as shown above, it was possible that cytokine activation in T cells from infected mice was mediated by superantigens associated with heat-killed bacteria. To address specificity of the recall response, B6 mice that were inoculated twice with GAS-2W streptococci and then were restimulated *in vivo* by intravenous injection of either HK-GAS-2W or heat killed wild-type 2W^-^GAS (HK-GAS-WT) bacteria. CD4^+^CD44^Hi^2W:I-A^b+^ cells from mice that were primed with GAS-2W had expanded significantly, produced IL-17A 21 days later following restimulation with HK-GAS-2W, but not with 2W^-^ HK-GAS-WT bacteria (Fig. 4C). Thus both priming of CD4^+^ T cells and recall of cytokine expression by GAS is antigen-specific and independent of superantigens in this animal model.

Children often experience multiple episodes of GAS pharyngitis before reaching the age of 15. Therefore, we investigated whether repeated infection amplified, shifted or dampened the Th17 response. Following multiple infections, most of the CD4^+^CD44^Hi^2W:I-A^b+^ T cells had elevated levels of intracellular IL-17A (Fig.4B). The response was more robust in NALT, the major infection site following intranasal GAS infection.

### Route of inoculation influences the Th17 phenotype to GAS infection

**Figure 5:**
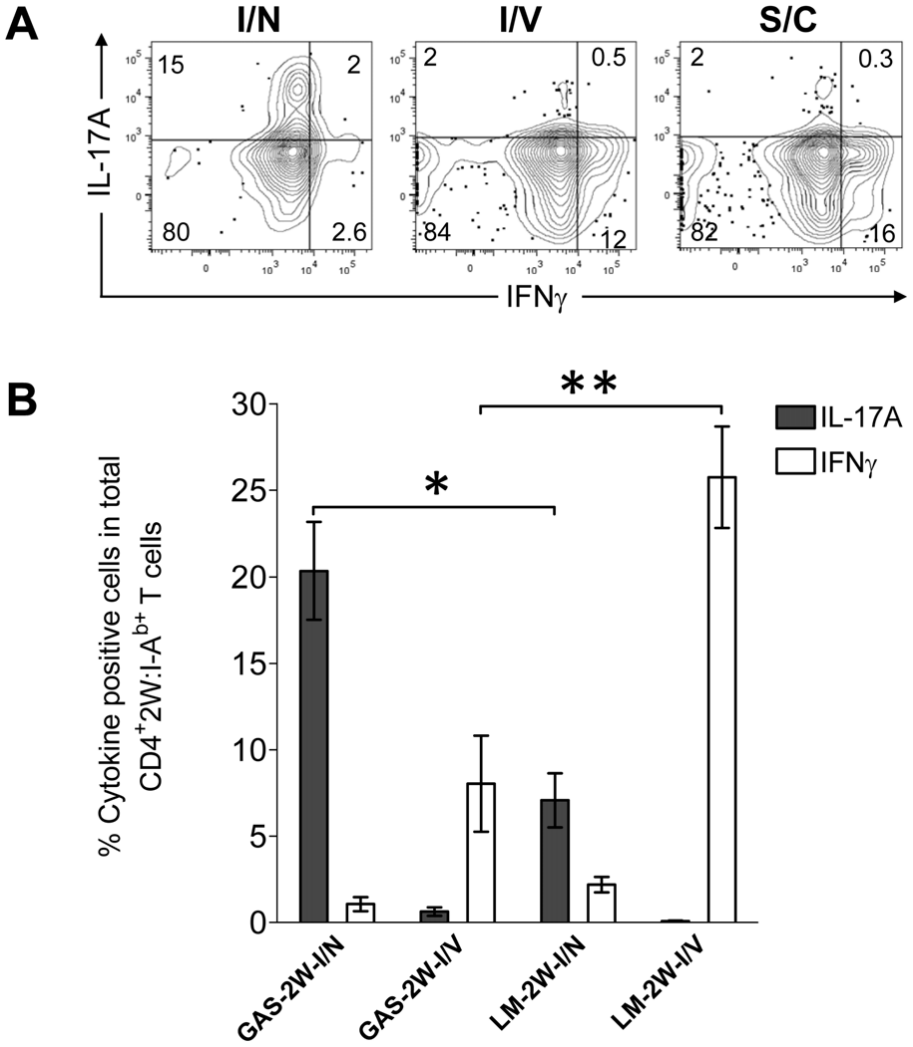
Route of inoculation determines the Th17 phenotype to GAS infection. (A) B6 mice were inoculated intranasally (I/N), intravenously (I/V) or subcutaneously (S/C) with HK-GAS-2W. After one week mice were restimulated in vivo with HK-GAS-2W for 4 hours and then CD4^+^CD44^Hi^2W:I-A^b+^ cells from spleen were stained for cytokines IL-17A and IFN-γ and analyzed by flow cytometry. (B) Comparison of the CD4^+^CD44^Hi^2W:I-A^b+^ T cell response to GAS-2W or *Listeria monocytogenes* (LM-2W) intranasal and intravenous infections. Three weeks after a single intranasal or intravenous inoculation with bacteria, single cell suspensions from spleens were incubated with PMA and ionomycin for 4 hrs. CD4^+^CD44^Hi^2W:I-A^b+^ cells were then enriched, stained and analyzed for IL-17A and IFN-γ expression by flow cytometry. (n = 7-12 mice per group). The bars represent the mean ± SEM. * =  P<0.002, **  =  P<0.001, Mann Whitney test.

Recently, Pepper *et al* showed that the intranasal route was important for the generation IL-17-producing 2W:I-A^b^-specific T cells in response to a recombinant strain of *Listeria monocytogenes* that expressed the 2W peptide (LM-2W) [20]. Most GAS infections naturally involve oro-pharyngeal tissue and preferably colonize tonsils in humans and NALT in mice. Experiments were therefore performed to assess the impact of the intranasal route of inoculation of GAS on the CD4^+^ T cell response. B6 mice were inoculated with HK-GAS-2W intranasally, intravenously or subcutaneously. HK-GAS-2W bacteria were used to avoid spread of infection from the site of inoculation. 2W:I-A^b^-specific T cells expanded in the spleens of these mice in response to all routes of inoculation. Intranasal infection with GAS-2W induced IL-17A-producing 2W:I-A^b^-specific T cells; whereas intravenous and subcutaneous inoculations induced IFN-γ-producing cells (Fig. 5). The Th17/Th1 ratio was more than 40 times higher in cells from intranasally inoculated mice compared to that from mice inoculated intravenously or subcutaneously. Thus, the intranasal route of infection was critical for the generation of Th17 cells during GAS infection.

To compare the relative magnitude of Th17 induction by GAS and LM intranasal infections, mice were inoculated either intranasally or intravenously with either GAS-2W or LM-2W. Intranasally both GAS-2W and LM-2W induced primarily a Th17 response. Notably, however, a larger fraction of the 2W:I-A^b^-specific T cell population produced IL-17A following intranasal GAS-2W infection than following intranasal LM-2W infection (Fig. 5B) [20] ; Furthermore, both GAS and LM primarily induced a Th1 response to intravenous inoculation but LM induced a significantly greater Th1 response than GAS (Fig. 5B). Therefore, the PAMPs expressed by or nature of GAS infections creates an environment more conducive to priming the Th17 phenotype than those of LM. Moreover, these data suggest that the 2W epitope does not influence the Th phenotype per se, since the same naïve 2W:I-A^b^-specific CD4^+^ T cell population has expanded and differentiated into different phenotypes in response to alternative routes of infection.

### IL-6-deficient mice fail to develop a Th17 response and to clear Group A Streptococci from NALT

**Figure 6:**
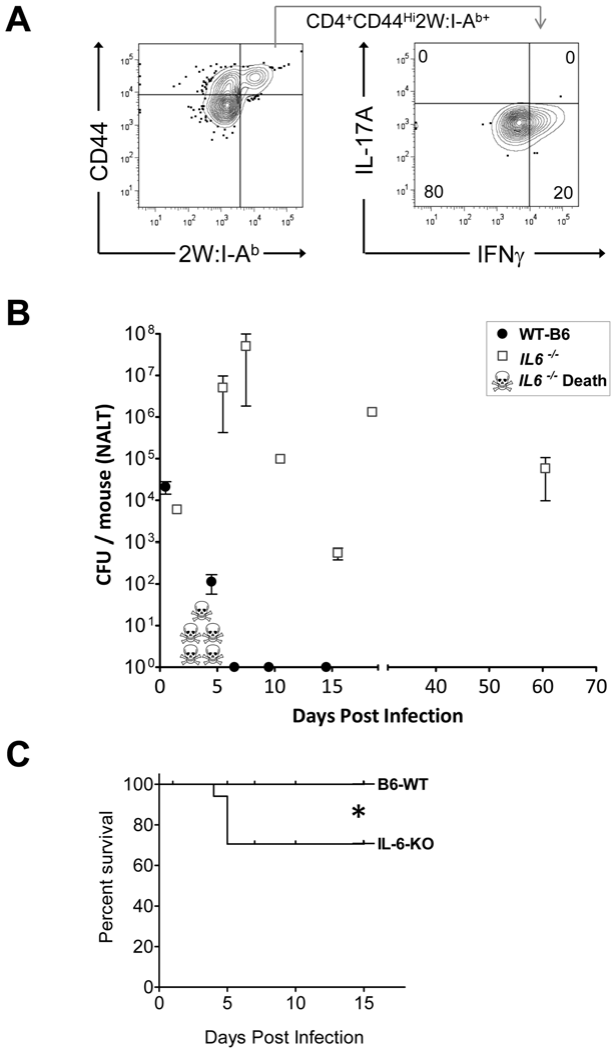
Mice deficient in Th17 cells fail to clear primary GAS infections from NALT and become chronic carriers. (A) Analysis of IL-17A and IFN-γ expression of CD4^+^CD44^Hi^2W:I-A^b+^ T cells from IL-6 knockout mice (*IL-6* ^-/-^) infected with GAS-2W. CD4^+^CD44^Hi^2W:I-A^b+^ cells were enriched from spleens of *IL-6* ^-/-^ mice that were intranasally inoculated with HK-GAS-2W and restimulated in vivo after a week by IV injection of HK-GAS-2W. (B) Comparison of *IL-6* ^-/-^ mice (n = 17) to age matched wild type B6 mice (n = 14) for susceptibility to intranasal GAS infection. Mice were inoculated with 2×10^8^ CFU/mouse intranasally. Mice were euthanized at different time points after inoculation and CFU associated with NALT were assessed. Data are presented as mean CFU ± SEM. (C) Kaplan-Meier survival curve of *IL-6^-/-^* and WT-B6 mice (*P<0.04, Logrank Test.)

Although IL-6 is known to be critical for some Th17 responses in mice, it is not known whether this is the case for streptococcal infection. We therefore compared the primary T cell response to GAS infection of B6 IL-6 knockout mice *(IL-6 ^-/-^)* mice to age matched wild type B6 mice, inoculated intranasally with 2x10^8^ cfu of GAS-2W. There was no significant difference in the colonization of both groups of mice 24 hrs after inoculation with this sublethal dose of bacteria. However, over the next 7 days nearly a third of the *IL-6 ^-/-^* mice succumbed to infection (*P = 0.0473, Logrank Test) (Fig. 6). In contrast, wild-type B6 mice had no casualties and most had reduced their bacterial load in NALT. *IL-6^-/-^* mice that survived infection continued to harbor high bacterial counts for up to 60 days; whereas, all wild-type mice completely cleared the bacteria from NALT by day 7.

*IL-6^-/-^* mice that survived the first week of infection returned to health even though they retained significant numbers of GAS-2W organisms in NALT. T cells from both wild-type and *IL-6* ^-/-^ mice were restimulated *in vivo* after one week by intravenous injection of HK-GAS-2W. The 2W:I-A^b+^ cells from *IL-6* ^-/-^ mice (Fig. 6A) had proliferated in response to infection with GAS-2W streptococci but failed to produce IL-17A. Instead, many of these cells produced IFN-γ (Fig. 6A). These results confirm that IL-6 is required to promote a Th17 response after GAS infection. Long-term persistence of GAS-2W in NALT from *IL-6^-/-^* mice is consistent with the possibility that clearance from NALT and perhaps human tonsils requires a vigorous Th17 adaptive response and that IFN-γ^+^ T cells lack potential to eliminate streptococci from lymphoid tissue.

### Recurrent GAS infection shifts the antigen-specific population toward an IL-17A^+^ IFN-γ^+^ double positive phenotype in NALT

**Figure 7:**
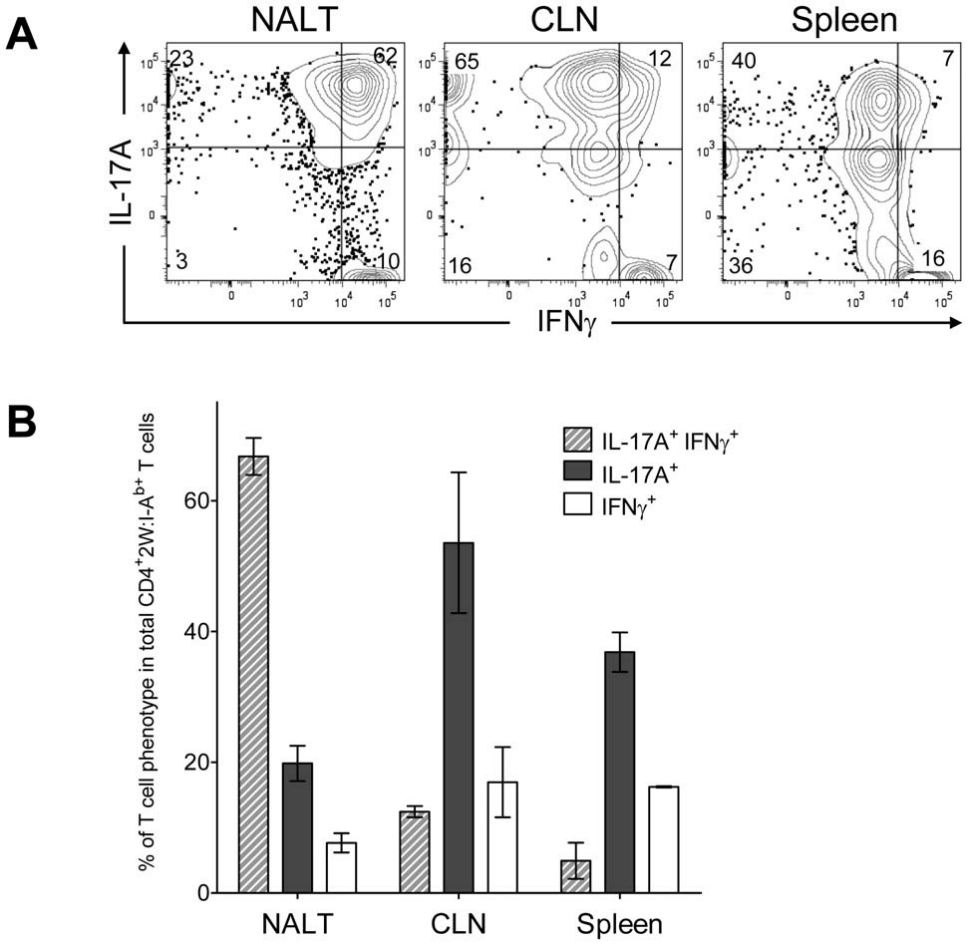
Recurrent GAS infection shifts the cytokine profile of CD4^+^ 2W:I-A^b+^ T cells to IL-17A^+^, IFN-γ^+^ population in NALT. (A) B6 mice were inoculated intranasally with 2×10^8^ CFU at weekly intervals for 12 weeks. CD4^+^CD44^Hi^2W:I-A^b+^ T cells from Spleens, CLNs and NALT of infected mice were restimulated in vitro with PMA and ionomycin, stained and analyzed for IL-17A and IFN-γ and analyzed by flow cytometry. Representative result from one of two independent experiments is shown. (B) Pooled results of two independent experiments shown. (n = 4).

The Th17 phenotype is less stable than the Th1 phenotype [20], [22], [23]. For example, IL-17A^+^ T cells were observed to acquire the capacity to produce IFN-γ *in vitro* by varying the cytokine environment [24]. The regulatory program and precursor to these double positive T cells is unclear, as is the impact of infection on this phenotype. These considerations prompted experiments to test the influence of multiple intranasal infections on the Th17 population in NALT. Wild-type B6 mice usually clear GAS from NALT 5-7 days after an intranasal inoculation [25]. For recurrent infection, B6 mice were inoculated intranasally with 2×10^8^ GAS-2W CFU at weekly intervals for 12 weeks. One week after the last inoculation cells were harvested from NALT, CLN and spleen, activated *in vitro* with PMA and ionomycin, and analyzed for intracellular cytokines IL-17A and IFN-γ. After repeated exposure to GAS-2W, IL-17A^+^ IFN-γ^+^ 2W:I-A^b^-specific CD4^+^ cells preferentially accumulated in the NALT, whereas the primary phenotype of CD4^+^CD44^Hi^2W:I-A^b+^ cells in spleen and CLN from the same mice retained a Th17 phenotype (Fig. 7A and 7B). More than 60% of CD4^+^CD44^Hi^2W:I-A^b+^ cells were positive for both cytokines; whereas, fewer than 12% from spleens and cervical lymph nodes were double positive. The implications of this change will be discussed below.

## Discussion

We recently discovered that GAS induces a polyclonal, TGF-β1 dependent Th17 response in intranasally infected mice. Moreover, adoptive transfer of CD4^+^ IL-17A^+^ T cells imparted partial protection to naïve mice against intranasal infection. That investigation raised several questions, which could be more definitively answered using pMHCII tetramers to quantify antigen specific T cells that recognize a streptococcal expressed antigen. In lieu of identifying immunodominant bacterial specific T cell epitopes, the surrogate 14 amino acid peptide 2W was fused to M1 protein and expressed on the streptococcal surface. The recombinant serotype M1 strain, GAS-2W, was constructed and M protein function confirmed by phagocytosis assays using human blood. Moreover, the GAS-2W strain colonized mouse NALT as efficiently as wild type 90-226 streptococci.

For unknown reasons, humans often fail to develop protective immunity after a single episode of pharyngitis or tonsillitis, and persistence of GAS in tonsils even after antibiotic intervention is common [7]. This problem could be explained by failure to develop a sufficient Th17 immune response. However, we found that a single intranasal infection with GAS-2W rapidly induced a robust 2W:I-A^b^-specific Th17 population, which paralleled the clearance of bacteria in mice. The 2W:I-A^b^-specific Th17 population represented ∼10% of the total GAS specific Th17 induced (Fig. S1). Superantigens are powerful mitogens that induce massive TCR V beta chain-specific clonal expansion of T cells. Group A streptococci, including the GAS-2W strain are known to produce many superantigens, which have been implicated in highly lethal streptococcal toxic shock and autoimmune sequelae [1], [26]. Such superantigens do not account for the expansion of 2W:I-A^b^-specific CD4^+^ T cells following GAS-2W infection, despite the fact that some of these T cells express the relevant TCR V beta chains [19]. This conclusion may not reflect the situation during human infections because murine T cells are known to be relatively refractory to bacterial superantigens. Dominance of the Th17 response to GAS is impressive relative to that observed for other bacterial pathogens like Listeria. After a single infection 20-39% of the 2W-specific T cells produced IL-17A. By comparison, only about 5–12% of 2W:I-A^b^-specific CD4^+^ cells produced IL-17A in response to primary LM-2W intranasal infection (Fig. 5, [20]). Furthermore, both GAS and LM primarily induced a Th1 response to intravenous inoculation and as expected LM induced a more robust Th1 response than GAS (Fig. 5B). Therefore, GAS might have other features that make for more efficient Th17 induction than LM. Furthermore, these results suggest that the 2W epitope per se does not influence the out come of the CD4^+^ T cell response. The more robust Th17 response could reflect the GAS tropism for NALT [8] and human tonsils [4], the potential for these bacteria to induce TGF-β1 production in these sites [10], expression of a unique pathogen-associated molecular patterns (PAMPs), or a combination of these factors. GAS was shown to induce TGF-β1 and IL-6 in NALT [10], but the specific PAMPs that lead to expression of these cytokines are unknown. The substantial hyaluronic acid capsule or its degradation products are known to induce TLR2- and TLR4-mediated inflammation [27], [28], and TLR2 agonists are known to promote Th17 differentiation [29]. M1 protein is also a TLR2 agonist [30] and known to induce inflammatory cytokines, including IL-6 [31] and TGF-β1 [32], and is, therefore, another potential PAMP that could direct a Th17 response. The finding that intranasal inoculation, even with heat-killed GAS-2W cells induces an antigen-specific Th17 response, while other routes induce a Th1 response also has important implications for development and delivery of vaccines to protect against GAS and other mucosal pathogens. The pathogenic potential of IL-17A^+^ T cells and their short half-life questions the potential safety and utility of intranasal vaccines.

We previously observed that single cell suspensions of NALT, spleen, and cervical lymph nodes from intranasally infected mice secrete TGF-β1, IL-6 and IL-17 upon *ex vivo* restimulation [10]. Although IL-17 secretion was dependent on TGF-β1 receptor signals, dependence on IL-6 was not tested. As predicted [33], *IL-6^-/-^* mice failed to mount a Th17 response and instead developed IFN-γ^+^ T cells following intranasal infection. As reported by Diao *et al*, we found high mortality in *IL-6^-/-^* mice infected with GAS [34], perhaps as a consequence of exuberant TNF-α expression. Nearly a third of *IL-6* ^-/-^ mice, inoculated intranasally, developed lethal systemic infections; however, survivors fully recovered without subsequently developing systemic infections even though they retained large numbers of GAS in NALT (Fig. 6). This suggests that protection is compartmentalized, ie. opsonic antibody is required to clear bacteria from blood, but does not efficiently remove them from NALT or tonsils. On the other hand a robust Th17 cellular response would be required to eliminate streptococci from those lymphoid organs. Secretory antibody that baths mucosal surfaces may also protect against pharyngitis, but may not be able to reach bacteria sequestered within NALT. These findings are also consistent with studies of Th17 protection induced by intranasal infections of mice with encapsulated *S. pneumoniae* [11]. It is theoretically possible that local activation of antigen specific T cells could recruit phagocytes with potential to ingest other unrelated bacteria at that site, but recruitment of phagocytes to those infectious foci would still be antigen specific. In addition, *IL-6^-/-^* mice developed a robust antigen specific Th1 response (Fig. 6) but failed to clear the bacteria from NALT. This finding is consistent with the observation that *IFN-γ^-/-^* mice [35] cleared streptococci from NALT more rapidly than normal mice. We postulate that efficient elimination of GAS depends on IL-17A production by Streptococcal specific CD4^+^ T cells to attract neutrophils to infected NALT, a capacity that IFN-γ-producing T cells lack. Persistence of large numbers of GAS in NALT of *IL-6^-/-^* mice is reminiscent of the situation in human immune carriers who may also fail to develop an adequate Th17 response.

The Th17 response is known to contribute to immune protection against infections by several pathogens [10], [11], [36], [37], [38]; however, these T cells can also be pathogenic, as demonstrated in murine autoimmune models, such as experimental encephalomyelitis (EAE) [39], [40]. A potentially important discovery from our experiments is that repeated intranasal exposure to GAS-2W results in the accumulation of an IL-17A^+^ IFN-γ^+^ (double positive) 2W:I-A^b^-specific CD4^+^ T cells in NALT. In contrast, 2W:I-A^b^-specific CD4^+^ T cells in CLN and spleens from the same mice predominantly retained the Th17 phenotype. Preferential accumulation of double positive T cells in NALT could be explained by a unique homing potential, by more rapid expansion or a longer half-life of double positive cells relative to those only able to produce IL-17A. Whether the large population of antigen-specific IL-17^+^ IFN-γ^+^ double positive cells observed in NALT following recurrent GAS infection is associated with an autoimmune response is unknown: however, repeated intranasal infection of mice was reported to occasionally produce endocarditis and valvular lesions[41], suggesting a link to human autoimmune disease.

## Methods

### Ethics statement

This study was carried out in strict accordance with the recommendations in the Guide for the Care and Use of Laboratory Animals of the National Institutes of Health. All animal experiments were conducted under University of Minnesota Institutional Animal Care and Use Committee (IACUC) approved protocol number 0806A36362.

### Bacterial strains and growth

Streptococci were grown in Todd-Hewitt broth supplemented with 2% neopeptone (THB-Neo) or on solid media containing Difco blood agar base and sheep blood at 37°C in 5% CO_2_. All growth media were purchased from Difco Laboratories, Detroit, MI. Strain 90–226 (serotype M1) was originally isolated from the blood of a septic patient [18].

### Mice

Five to six weeks old female C57BL/6 mice were purchased from Taconic farms (Germantown, NY) and used at 7–8 weeks of age. *IL-6* ^-/-^ mice in C57BL/6 background were purchased from the Jackson Laboratory (Bar Harbor, ME) [42]. Mice were housed under specific pathogen-free conditions at RAR facilities of University of Minnesota. Mice inoculated with GAS were housed in biosafety level 2 facilities.

### Generation of a recombinant GAS strain that expresses the 2W epitope in M protein

An M1 GAS strain 90–226 was genetically engineered to express the 14 amino acid 2W peptide (EAWGALANWAVDSA) on the bacterial surface as a fusion protein with streptococcal M protein (Fig. 1). The corresponding chimeric protein is composed of the 2W epitope inserted in-frame after the first five amino acids of the mature M1 surface protein. The hybrid emm1.0::2W gene was constructed using standard molecular biology techniques and then introduced into the chromosomal emm1.0 gene locus by allelic replacement. Briefly, the emm1.0 gene of strain 90–226 is contiguous with mga at the 5′ end and with the sic gene at the 3′ end, and has its own promoter. Plasmid pFW5 has two multiple cloning sites on either side of a spectinomycin resistance gene [8], [18]. The entire sic gene with its promoter was PCR amplified and inserted into the multiple cloning site downstream of the spectinomycin resistance gene in pFW5. A fragment containing the C-terminal half of mga through the entire emm1.0 gene was PCR amplified by a two-step method to insert the sequence of the 2W epitope into the emm1.0 gene. In the first step, DNA fragments, each coding for part of the 2W epitope sequence (overlapping) were amplified by PCR. In the second step these overlapping fragments were annealed, extended and further amplified to generate a 2.5-kb fragment, which was inserted into the multiple cloning site upstream of the spectinomycin resistance gene in pFW5. This plasmid was transformed into strain 90–226 emm1.0::km for gene replacement, and transformants were selected on spectinomycin (100 µg/ml). The spectinomycin-resistant transformants were then screened for kanamycin sensitivity. Positive clones (Spec^R^Kan^S^) were screened for gene replacement and the entire region including the 2W insertion was amplified and sequence verified. The resulting strain was designated *Streptococcus pyogenes* 90-226 emm1.0::2W (GAS-2W). A whole blood phagocytosis assay was used to test whether GAS-2W streptococci are M^+^ [43].

### Intranasal inoculation

C57BL/6 mice were anesthetized with isoflurane/oxygen mixture for 1 min and inoculated intranasally with GAS-2W by placing appropriate doses in a total volume of 15 µl PBS (7.5 µl/nostril). Fractionated droplets were placed with a pipette tip near the entrance of the nostril and the inoculum was involuntarily aspirated into the nasal cavity.

### In vitro stimulation with PMA-ionomycin

NALT, Cervical lymph nodes (CLN) and Spleen from C57BL/6 mice were harvested by dissection, disrupted on a nylon screen in 1–2 ml of complete EHAA medium (cEHAA). Resulting single cell suspensions were filtered over a nylon screen and washed with cEHAA before invitro stimulation and staining. Freshly made single cell suspensions in cEHAA were stimulated in vitro with PMA and ionomycin for 4 hours. PMA and ionomycin were added at 50 ng/ml and 500 ng/ml final concentrations, respectively. Cells were incubated at 37C for 1 hour and Brefeldin A (BrA) was added to disrupt golgi and prevent cytokines from being secreted into the medium (1∶1000 of 10 mg/ml DMSO stock) and incubated for three additional hours before proceeding to tetramer enrichment.

### Tetramer production

2W:I-A^b^ tetramers were generated as described in detail by Moon *et al* [16], [19]. Briefly, 2W:I-A^b^ was expressed in S2 cells. S2 cells were co-transfected with plasmids encoding the I-A^b^ alpha chain, the I-A^b^ beta chain and BirA gene. Transfected cells were selected, passaged in serum-free media, and cultures scaled up to one liter cultures. Expression was induced with copper sulfate and biotinylated 2W:I-A^b^ heterodimers were purified from supernatants with a Ni^++^ affinity column. Bound 2W:I-A^b^ molecules were eluted and purified. Tetramers were created by mixing biotinylated 2W:I-A^b^ molecules with Phycoerythrin (PE) or Allophycocyanin (APC)-conjugated streptavidin.

### Peptide-MHC-II tetramer based magnetic bead enrichment

2W:I-A^b^-specific CD4^+^ T cells were enriched as described previously [16], [19], [20]. Briefly mice were euthanized and NALT, cervical lymph nodes and Spleen were harvested separately. Single cell suspensions were prepared in 200 µl of Fc block (supernatant from 2.4G2 hybridoma cells grown in serum-free media + 2% mouse serum, 2% rat serum, 0.1% sodium azide) from each tissue. Phycoerythrin (PE) or Allophycocyanin (APC) conjugated tetramer was added at a final concentration of 10 nM and the cells were incubated at room temperature for 1 hr, followed by a wash in 15 ml of ice-cold FACS buffer (PBS + 2% fetal bovine serum, 0.1% sodium azide). The tetramer-stained cells were then resuspended in a volume of 200 µl of FACS buffer and mixed with 50 µl of anti-PE or anti-APC antibody conjugated magnetic microbeads (Miltenyi Biotech, Auburn, CA) and incubated on ice in dark for 30 min, followed by one wash with 10 ml of FACS buffer. The cells were resuspended in 3 ml of FACS buffer and passed over a magnetized LS column (Miltenyi Biotech). The column was washed with 3 ml of FACS buffer three times and then removed from the magnetic field. The bound cells were eluted by pushing 5 ml of FACS buffer through the column with a plunger. The resulting enriched fractions were resuspended in 0.1 ml of FACS buffer, and a small volume was removed for cell counting while the rest of the sample was stained with a cocktail of fluorochrome-labeled antibodies.

### In vivo stimulation with heat-killed GAS-2W

GAS-2W was grown in THB-Neo media to O.D._600_ of 0.5, washed once with PBS, pelleted and resuspended in appropriate volume of PBS and incubated at 60C for 30 minutes. Viability of bacteria was confirmed by plating out on blood agar plates. Heat-killed GAS-2W was stored in aliquots at -20C until use. To induce cytokine production by 2W:I-A^b^-specific CD4^+^ T cells in vivo, 2 X 10^8^ CFU heat-killed GAS-2W in 200 µl of PBS was inoculated intravenously through tail vein. Mice were sacrificed after 3–4 hrs and single cell suspensions of spleen were made in cEHAA medium supplemented with BrA.

### Intracellular cytokine staining

Intracellular cytokine staining for IL-17A and IFN-γ was done using BD Cytofix/Cytoperm fixation-permeabilization solution and anti-IL-17A-PE and anti-IFN-γ-PE-Cy7 antibodies according to manufacturer's protocols. Briefly cells stimulated in vivo with heat-killed GAS-2W or in vitro with PMA-ionomycin were subjected to 2W-MHCII tetramer enrichment. Enriched cells were stained for surface markers, washed and fixed with 250 µl of Fixation/Permeabilization solution (BD Biosciences) per sample for 20 min on ice. All samples were washed with 1 ml of 1X Perm/Wash buffer (BD Biosciences) and anti IL-17A PE and anti IFN-γ-PE-Cy7 antibodies were added in 100 µl of 1X Perm/Wash buffer and incubated overnight at 4C in dark. The following day cells were washed with 1 ml of 1X Perm/Wash buffer and resuspended in 400 µl of FACS buffer and transferred to a micro tube within a FACS tube for analysis in LSR-II flow cytometer.

### Flow cytometry and antibodies

A Becton Dickinson LSR-II flow cytometer was used to collect and analyze events that have the light scatter properties of lymphocytes and are CD4^+^ and 2W:I-A^b+^. Pacific Blue-conjugated anti-B220, CD11b, F4/80 (Caltag), CD11c (eBioscience, San Diego, CA); Pacific Orange-conjugated CD8 (eBioscience); PerCP-conjugated CD4 (BD PharMingen); PE-Cy7-conjugated IFN-γ (eBioscience); PE conjugated IL-17A; APC-AF780 conjugated CD4 and AlexaFluor 700-conjugated CD44 (eBioscience); PE-conjugated CD4; FITC-conjugated CD4; PB-conjugated CD4; APC-conjugated CD4; AF700-conjugated CD4; PerCP-Cy5.5-conjugated CD4; APC-AF750-conjugated CD4. Antibodies were purchased from the indicated sources. FACS acquisition was performed using FACSDiva software and analysis was done using FlowJo (Tree Star, Ashland, OR).

### Statistics

All values are expressed as mean ± SEM. Differences between groups were analyzed using Student's t-test by GraphPad Prism (Version 4.03 for Windows, GraphPad Software, San Diego, CA). Differences are considered significant at P<0.05.

## Supporting Information

Figure S1 **Primary intranasal GAS infection induces a Th17 response in both 2W:I-A^b+^ and 2W:I-A^b-^ CD4^+^ T cell populations.** B6 Mice were inoculated once intranasally with 2×10^8^ CFU of GAS-2W. 10 days after the infection mice were restimulated in vivo with IV injection of heat killed GAS-2W. 2W:I-A^b+^ CD4^+^ T cells from spleen were enriched on magnetized columns. Both bound and unbound fractions were collected and analyzed for intracellular cytokines IL-17A and IFN-γ. Upper panel shows the bound fraction and cells shown in the middle and right columns are gated on CD4^+^CD44^Hi^ cells. Lower panels shows the unbound (flow through) fraction. One representative of two independent experiments is shown. Numbers of CD4^+^IL-17A^+^ T cells shown in the quadrants above were calculated for the whole spleen. The approximate ratio of 2W:I-A^b+^IL-17A^+^ cells to streptococcus activated total 2W:I-A^b-^ IL-17A^+^ cells ranged from 1:7 to 1:12.
(TIF)
