# Supplementary figures and images for: Robust Antigen Specific Th17 T Cell Response to Group A Streptococcus Is Dependent on IL-6 and Intranasal Route of Infection
Source: PLoS Pathog. 2011 Sep 22;7(9):e1002252. doi: 10.1371/journal.ppat.1002252 (PMC3178561; doi:10.1371/journal.ppat.1002252)

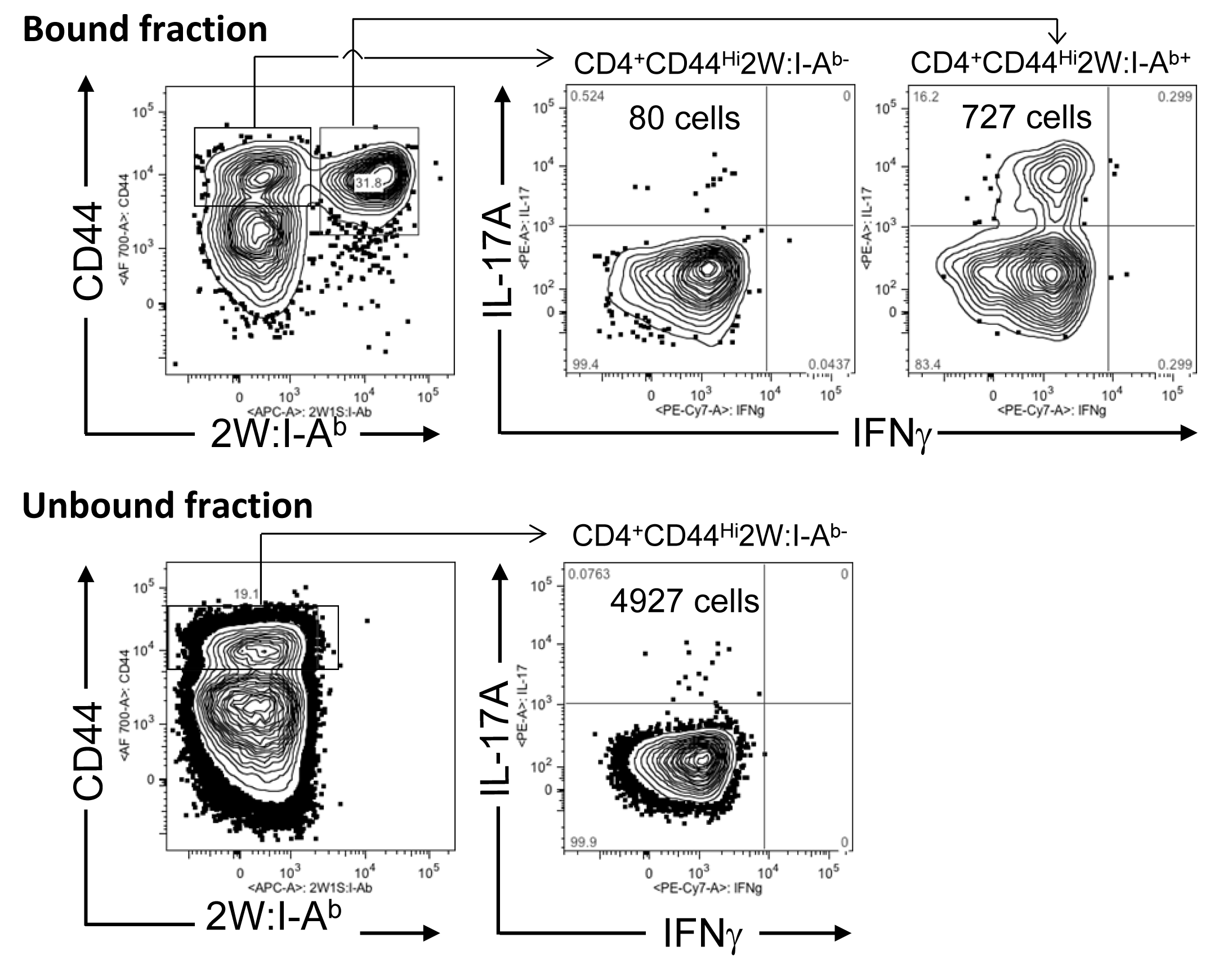

Supplement: Figure S1 — Primary intranasal GAS infection induces a Th17 response in both 2W:I-Ab+ and 2W:I-Ab- CD4+ T cell populations. B6 Mice were inoculated once intranasally with 2×10^8^ CFU of GAS-2W. 10 days after the infection mice were restimulated in vivo with IV injection of heat killed GAS-2W. 2W:I-Ab+ CD4+ T cells from spleen were enriched on magnetized columns. Both bound and unbound fractions were collected and analyzed for intracellular cytokines IL-17A and IFN-γ. Upper panel shows the bound fraction and cells shown in the middle and right columns are gated on CD4+CD44Hi cells. Lower panels shows the unbound (flow through) fraction. One representative of two independent experiments is shown. Numbers of CD4+IL-17A+ T cells shown in the quadrants above were calculated for the whole spleen. The approximate ratio of 2W:I-Ab+IL-17A+ cells to streptococcus activated total 2W:I-Ab- IL-17A+ cells ranged from 1:7 to 1:12. (TIF) [file ppat.1002252.s001.tif]
